# Generating synthetic high-resolution spinal STIR and T1w images from T2w FSE and low-resolution axial Dixon

**DOI:** 10.1007/s00330-024-11047-1

**Published:** 2024-09-04

**Authors:** Robert Graf, Paul-Sören Platzek, Evamaria Olga Riedel, Su Hwan Kim, Nicolas Lenhart, Constanze Ramschütz, Karolin Johanna Paprottka, Olivia Ruriko Kertels, Hendrik Kristian Möller, Matan Atad, Robin Bülow, Nicole Werner, Henry Völzke, Carsten Oliver Schmidt, Benedikt Wiestler, Johannes C. Paetzold, Daniel Rueckert, Jan Stefan Kirschke

**Affiliations:** 1 Department of Diagnostic and Interventional Neuroradiology, School of Medicine, Technical University of Munich, Munich, Germany; 2 Institut für KI und Informatik in der Medizin, Klinikum rechts der Isar, Technical University of Munich, Munich, Germany; 3 Institute for Diagnostic Radiology and Neuroradiology, University Medicine Greifswald, Greifswald, Germany; 4 Institut für Community Medicine, Abteilung SHIP-KEF, University Medicine Greifswald, Greifswald, Germany; 5 Professor of Visual Information Processing, Department of Computing, Imperial College London, London, United Kingdom

**Keywords:** Spine, Magnetic resonance imaging, Databases, Factual, Deep learning

## Abstract

**Objectives:**

To generate sagittal T1-weighted fast spin echo (T1w FSE) and short tau inversion recovery (STIR) images from sagittal T2-weighted (T2w) FSE and axial T1w gradient echo Dixon technique (T1w-Dixon) sequences.

**Materials and methods:**

This retrospective study used three existing datasets: “Study of Health in Pomerania” (SHIP, 3142 subjects, 1.5 Tesla), “German National Cohort” (NAKO, 2000 subjects, 3 Tesla), and an internal dataset (157 patients 1.5/3 Tesla). We generated synthetic sagittal T1w FSE and STIR images from sagittal T2w FSE and low-resolution axial T1w-Dixon sequences based on two successively applied 3D Pix2Pix deep learning models. “Peak signal-to-noise ratio” (PSNR) and “structural similarity index metric” (SSIM) were used to evaluate the generated image quality on an ablations test. A Turing test, where seven radiologists rated 240 images as either natively acquired or generated, was evaluated using misclassification rate and Fleiss kappa interrater agreement.

**Results:**

Including axial T1w-Dixon or T1w FSE images resulted in higher image quality in generated T1w FSE (PSNR = 26.942, SSIM = 0.965) and STIR (PSNR = 28.86, SSIM = 0.948) images compared to using only single T2w images as input (PSNR = 23.076/24.677 SSIM = 0.952/0.928). Radiologists had difficulty identifying generated images (misclassification rate: 0.39 ± 0.09 for T1w FSE, 0.42 ± 0.18 for STIR) and showed low interrater agreement on suspicious images (Fleiss kappa: 0.09 for T1w/STIR).

**Conclusions:**

Axial T1w-Dixon and sagittal T2w FSE images contain sufficient information to generate sagittal T1w FSE and STIR images.

**Clinical relevance statement:**

T1w fast spin echo and short tau inversion recovery can be retroactively added to existing datasets, saving MRI time and enabling retrospective analysis, such as evaluating bone marrow pathologies.

**Key Points:**

- *Sagittal T2-weighted images alone were insufficient for differentiating fat and water and to generate T1-weighted images*.
- *Axial T1w Dixon technique, together with a T2-weighted sequence, produced realistic sagittal T1-weighted images*.
- *Our approach can be used to retrospectively generate STIR and T1-weighted fast spin echo sequences*.

**Graphical Abstract:**

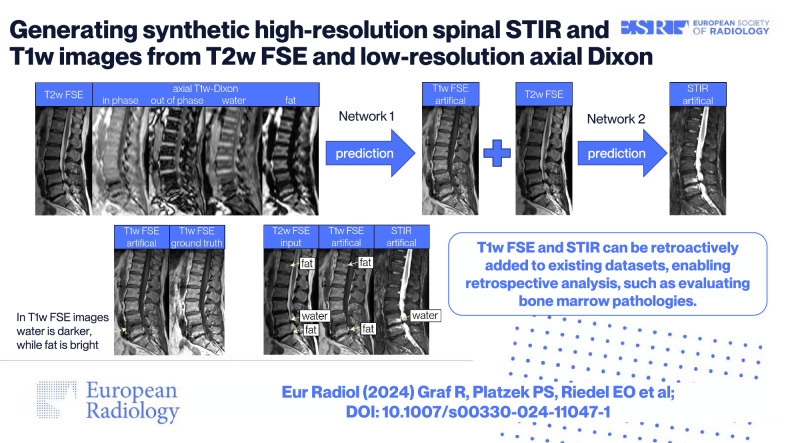

## Introduction

Magnetic resonance (MR) imaging plays a crucial role in diagnostic procedures. A major strength of MRI is its ability to acquire different imaging contrasts, enabling multi-faceted diagnostic assessment of various pathologies. The two most used sequences at the spine are T1-weighted (T1w FSE) and T2-weighted fast spin echo (T2w FSE). Several techniques have been developed to suppress either fat or water signals in MR scans, generating multiple images highlighting specific tissue characteristics [1, 2]. The MR acquisition itself is time-consuming, often necessitating the acquisition of 2D images with low through-plane resolution to reduce scan times and minimize the risk of subject movement, which could compromise image acquisition. Typically, two to four sequences encompassing different acquisition techniques and orientations are acquired, but the optimal set of required images may vary depending on the diagnostic context.

Artificial intelligence is emerging as a promising tool in the field of radiology. Many deep learning approaches rely on specific acquisition techniques and slice axes. Conditional generative networks can generate missing sequences [3–9], and it has been shown that with the matching MR sequences, fat-saturated images can be extracted [1, 2, 10–14], but not yet for T1w FSE sequences. For large-scale studies such as the German National Cohort (NAKO) [15], the “Study of Health in Pomerania” (SHIP) [16, 17], or the UK Biobank [18], rescanning subjects to obtain missing sequences is impossible. These studies commonly employ the axial T1-weighted gradient echo Dixon technique (T1w-Dixon; also, volume interpolated breath-hold examination) due to its versatility. However, its limited sagittal resolution renders it unsuitable for evaluating many spinal conditions. Only SHIP has T1w FSE images, and short tau inversion recovery (STIR) images are missing in all large studies. The NAKO and the SHIP have sagittal T2w FSE images, and the T1w-Dixon images contain the necessary information to decode water from fat [19, 20]. With those two data pairs, we aim to generate commonly used sagittal T1w FSE-like images that would greatly increase the usability of images in those large endemiological studies. Our technique can also be used to reduce scan time by not requiring additional scans.

It is crucial to differentiate between increased signal intensities in sagittal T2w FSE images caused by fat and water, e.g., as this can differentiate a chronic degenerative from an active inflammatory process [21–23]. STIR specializes in highlighting water content, enhancing diagnostic sensitivity [24–27], and reducing the risk of unintentionally overlooking water-related hyperintensities. Combining T1w and T2w FSE images makes it possible to distinguish between fat and water, as water appears dark in T1w FSE images while fat remains brighter compared to the surrounding tissue. This study’s objective is to assess, whether the acquisition of sagittal T2w FSE and axial T1w-Dixon sequences provides sufficient information to generate sagittal T1w FSE and sagittal STIR images in the same resolution as the T2w FSE.

## Materials and methods

The local ethics committee approved our research within existing legal frameworks for retrospective clinic internal data analysis. Informed consent for internal data collection was waived. All participants of the German National Cohort [15] (NAKO) and “Study of Health in Pomerania” [16, 17] (SHIP) studies signed informed consent for prospective data collection and evaluation.

While axial T1-weighted gradient echo Dixon technique (T1w-Dixon) contains information on fat and water content [19, 20], the resolution may not be sufficient to accurately assess water content in sagittal views. Therefore, to utilize axial T1w-Dixon similar to sagittal T1-weighted fast spin echo (T1w FSE), super-resolution of this data is necessary. We upscale and translate this data toward the already familiar modalities of T1w FSE and short tau inversion recovery (STIR). We use the sagittal T2-weighted (T2w) FSE image to guide the super-resolution process, ensuring that the networks utilize existing sagittal information from the T2w FSE image rather than extrapolating the structures. Thus, this study created sagittal STIR and T1w FSE volumes using sagittal T2w FSE and axial T1w-Dixon images. We employed two separate neural networks. The first network generated sagittal T1w FSE images from sagittal T2w FSE and axial T1w-Dixon images, trained on the SHIP dataset, consisting of 3165 subjects (1529 males, aged 53 ± 14 years (mean ± standard deviation); 1634 females, aged 53 ± 13 years, two others) on 1.5 Tesla MRI devices. While the sagittal FSE sequences covered the whole spine, the field of view of the T1w-Dixon images only spanned from the center of the chest to the sacrum.

The second network converted sagittal T1w FSE and T2w FSE images into sagittal STIR images based on the finding that these inputs contain sufficient biological information for STIR image reconstruction, as demonstrated in previous studies [2, 10–14]. Our internal dataset comprised 311 matching sequences, encompassing the entire spine, from 157 subjects (43 males, aged 54 ± 20 years; 45 females, aged 59 ± 16 years, 67 patients fully anonymized from a prior study, without age and sex available) with varying anomalies and pathologies. The internal data are acquired on either 1.5- or 3-Tesla MRI devices.

**Fig. 1:**
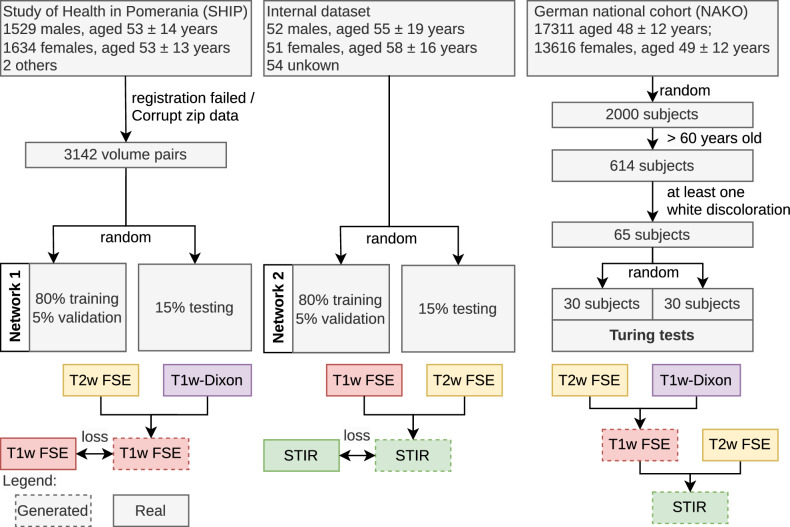
Datasets. We used the “Study of Health in Pomerania” for training the T1w generation from T1w-Dixon and T2w FSE images. The internal dataset is used for the STIR generation from T1w and T2w FSE images. For inference, we use images from the German National Cohort, where we found a fat or water uptick in the T2w FSE image. Below the dataset, the data flow is visualized, where dashed boxes indicate that they are generated by a deep learning network. STIR, sagittal short tau inversion recovery; T1w-Dixon, axial T1-weighted gradient echo Dixon technique; T1w, sagittal T1-weighted image; T2w, sagittal T2-weighted; FSE, fast spin echo image

Furthermore, the NAKO contributes axial T1w-Dixon and sagittal T2w FSE images from 30,927 subjects (17,311 males, aged 48 ± 12 years; 13,616 females, aged 49 ± 12 years) on 3-Tesla MRI devices. To demonstrate the applicability of our technique, we utilize a subset of the NAKO dataset, specifically selecting patients older than 60 years with at least one observable focal signal increase in the T2w images. These selection criteria were chosen to avoid an overabundance of normal images that could potentially obscure the evaluation of our method’s performance in identifying fat and water accumulations during Turing tests. The age threshold was chosen to speed up the selection process. An overview of our data pipeline can be found in Fig. 1.

### Image preprocessing

We reorient and rescale the T2w FSE images to a standardized resolution and orientation (Right, Inferior, Posterior). We adopt a sagittal in-plane resolution of 1.1 × 1.1 mm from the sagittal SHIP images. We maintain the original resolution for the right/left dimension of around 3–4 mm. The data are saved into multiple chunks along the vertical direction, which do not match between T1w-Dixon and the splits of the other image modalities. Current freely available software assumes that the sagittal images are on the same vertical *z*-axis [7], which is not true for scoliosis cases, where chunks follow the spine shape. To address this, we have developed software that computes the optimal bounding box encompassing all these chunks and stitches them together.^1^ The data chunks are already correctly encoded in global space. We address overlapping regions through interpolation $1-\frac{{d}_{x}}{{\sum }_{i}{d}_{i}}$, with the distance to the next voxel where no interpolation is necessary denoted by ${d}_{i}$ of image i. This ensured a smooth transition between the two images. The T1w-Dixon images were shifted by a few voxels compared to the T2w FSE image. Rigid registration with cross-correlation was applied with nipy-plugin to reduce the shift [28]. Black-box registration is not reliable for torso and spine registration due to breathing-, organ- and inter-vertebra movement. Registration reduced the shift but did not fully eliminate it, and in 18 cases, the registration failed fully. Those cases were excluded from the dataset. We resampled the T1w-Dixon data to match the voxel resolution of the adjacent T2w FSE image.

MR image values typically range from 0 to a maximum value, which can vary between different scan runs. We rescaled the image values from [0, max-value] to the range [−1, 1] to standardize this variability. Additionally, we applied random color jitter with a brightness and contrast-factor change ranging from 0.8 to 1.2 during training to account for the histogram inconsistencies. For training purposes, we used fixed input sizes of (16, 192, 192) pixels for T1w FSE generation and (16, 256, 256) pixels for STIR generation. During inference, we can insert the full image after padding it to be divisible by 8 in all image directions. To artificially increase the training data variance, we replicated the training data three times and resampled them to random sagittal resolutions between 1.0 and 0.5 mm.

### Image-to-image network

**Fig. 2:**
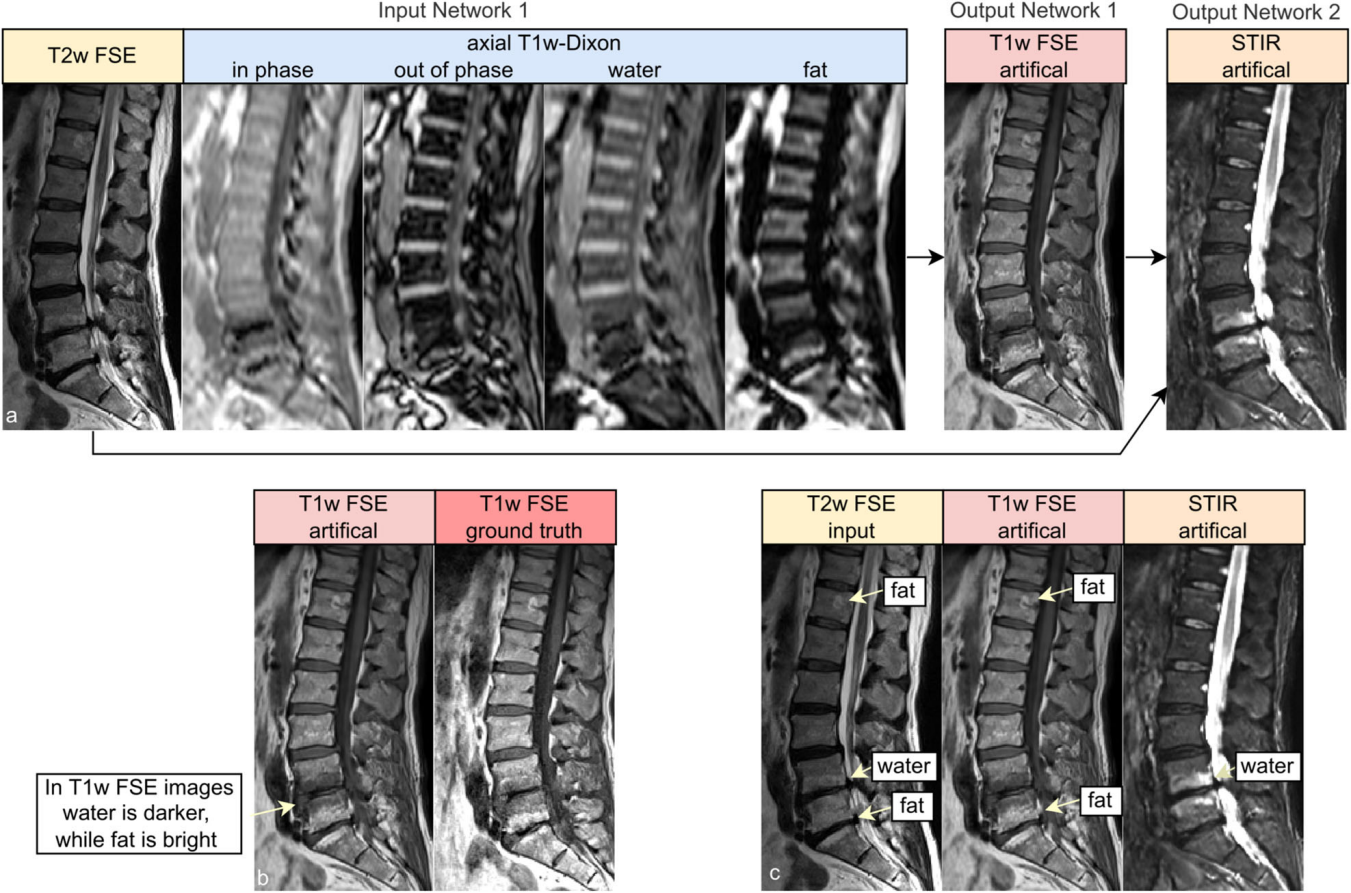
Sagittal views of input and output data. The image is from a participant of the “Study of Health in Pomerania” with Modic changes in L4/5 and L5/S1. **a** Example input and output of networks 1 and 2. The last vertebra contains a high-water content adjacent to L5/S1 (Modic 2), while it has high fat adjacent to L4/5 (Modic 1). The high-fat content can be seen in the T1w-Dixon fat image, while the high-water content is more difficult to distinguish. Network 2 receives the original T2w FSE image and the generated T1w FSE output of network 1. **b** A comparison of the generated and real image. The bone structures, the fat, and water aggregation are generated correctly. Only the stomach area differs because time has passed between the real T1w and T2w FSE image due to breathing, the digestive process, and filling their bladder during the examination. **c** Comparison of expression of water and fat in T1w, T2w FSE, and STIR images. In the STIR, the different hydronation of the inter-vertebra disks is easier to see than in the T2w FSE. STIR, sagittal short tau inversion recovery; T1w-Dixon, axial T1-weighted gradient echo Dixon technique; T1w, sagittal T1-weighted image; T2w, sagittal T2-weighted; FSE, fast spin echo image

For translation, we used the Pix2Pix [29] training mechanism. It uses a fully convolutional U-Net [30] (See Fig. 2) that takes an image volume stack as input and produces the target output. The reconstruction error was computed from three criteria: The absolute difference ${{{{\mathscr{L}}}}}_{1}$, the structural similarity index measure (SSIM) loss, and a least squares generative adversarial network (GAN) loss ${{{{\mathscr{L}}}}}_{2}$. The ${{{{\mathscr{L}}}}}_{1}$ and SSIM error is weighed by a factor of 10 to 1 toward the GAN loss.

${{{{\mathscr{L}}}}}_{{total}} \, =10 \, \cdot \, {{{{\mathscr{L}}}}}_{1}\left(\hat{x}-x\right) \, + \, 10 \,\cdot \, \left(1-{{{\rm{SSIM}}}}\left(\hat{x}-x\right)\right) \, +{{{{\mathscr{L}}}}}_{2}\left(D\left(\hat{x}\right),1\right)$ where $\hat{x}$ is the predicted volume, x is the ground truth, and D is a discriminator network. For the GAN loss, a patch-based discriminator is trained, and we followed the least squares GAN loss computation [31]. ${L}_{D}=0.5\cdot {{{{\mathscr{L}}}}}_{2}\left(D\left(\hat{x}\right),0\right)+0.5\cdot {{{{\mathscr{L}}}}}_{2}\left(D\left(x\right),1\right)$

**Fig. 3:**
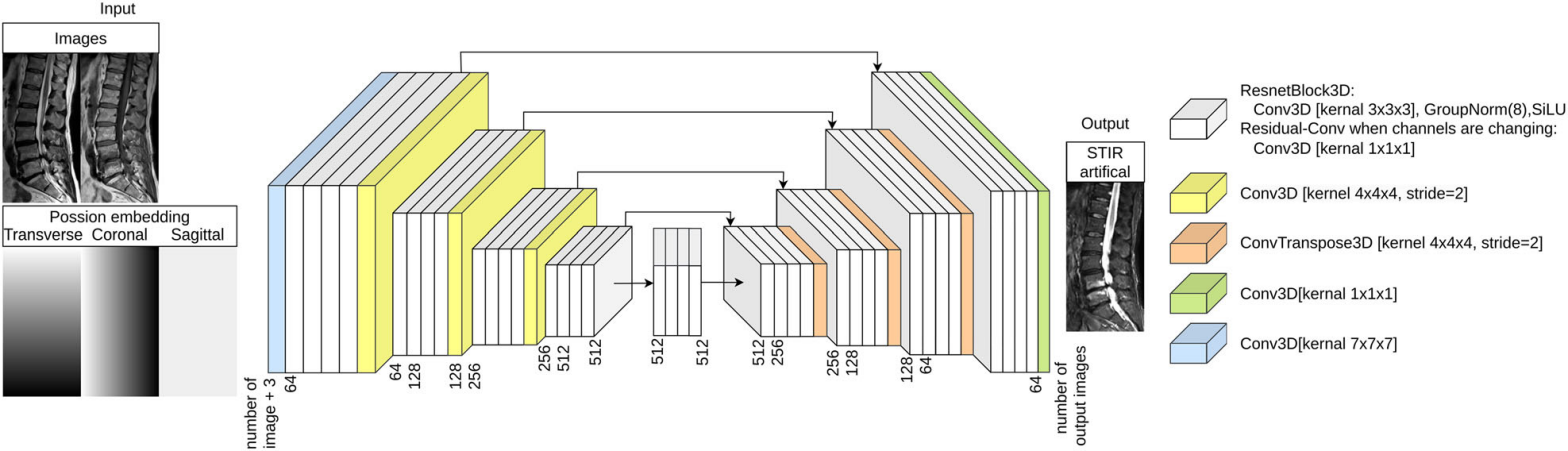
The network. We use 3D convolution, a group norm of 8, and a Sigmoid Linear Unit (SiLU) as the main residual block. The used channel sizes are indicated in the graphic and are changed at the first layer of the residual block. The model input (left) consists of the concatenation of all input images and the three-image position embeddings. The network produces the 3D target images as an output. The example shows network two in- and outputs

It is generally easier to train 2D models, but it comes at a loss of the additional information of the other slices. To stabilize the training and reduce the GPU storage for 3D Pix2Pix models, we incorporated some changes inspired by Bieder et al [32]. We used image embedding as three additional inputs to the image input stack. Each embedding is a gradient from 0 to 1 defined on the original borders of the 3D images before random cropping to the fixed patch size. Each volume dimension gets its own embedding gradient. See Fig. 3. We use addition instead of concatenation for skip-connections to reduce the GPU storage requirement. We train on a single V40 GPU with a learning rate of 0.00002 and a batch size of 3.

### Image quality

We aim to demonstrate the necessity of our approach for achieving optimal results. We evaluated our approach based on three criteria: (1) comparing the effectiveness of our 3D approach against 2D, (2) assessing the indispensability of utilizing both input pairs and (3) confirming the requirement of a paired setting. For (1), we employed the same network in 2D as in 3D Pix2Pix but increased the batch size to 64 and added attention layers to fully leverage the graphics card and optimal parameters for the 2D training. In (2), we compared the performance of both 3D and 2D networks using two inputs compared to only the T2w FSE image. To demonstrate the essentiality of data pairs (3), we employ 2D CycleGAN [33] on the same dataset and network. We use generated images and compare them to acquired images with absolute difference (L1) mean squared error (MSE), peak signal-to-noise ratio (PSNR), and SSIM.

### Turing test

A Turing test is designed to assess the ability of artificial intelligence to convincingly emulate authentic human behavior or data to deceive a human observer. In our study, we engaged seven radiologists with a set of 2D images comprising genuine T2w images and potentially generated images of another modality. The seven radiologists (P.S.P., E.O.R., S.H.K., N.L., C.R., K.J.P., O.R.K.—one radiologist with 3 years of experience; two each with 1, 2 and 6 years of experience) are tasked with distinguishing whether the adjacent image is authentic or artificially generated. We conducted this assessment separately for T1w FSE and STIR images. For T1w FSE, we employed 60 natively acquired images, with half originating from the SHIP dataset and the other half from our internal dataset. Additionally, 30 synthetic images were generated from each SHIP and NAKO dataset. In the case of the STIR Turing test, we produced 30 synthetic images from both the SHIP and NAKO datasets, while 60 authentic STIR images were extracted from our internal dataset. We exclusively utilized artificial T1w FSE images to generate synthetic STIRs in this test. Our objective was to maintain an equal balance between authentic and synthetic images. All images were standardized to a uniform size of 150 × 150 pixels with an in-plane resolution of 1.1 mm per pixel. We report the misclassification rate (false / (false + correct)) and Fleiss kappa interrater agreement test [34].

### Statistical analysis and software

The model analysis was performed with the Pytorch Python packages for the model performance. The *p*-values were computed with the Wilcoxon signed-rank test from the scipy Python packages. The Turing test and Fleiss kappa test were analyzed in Excel. The 99% confidence error for Fleiss kappa is calculated according to Cohen et al [34]. The Fleiss kappa has the interpretation of 0–0.2 Poor, 0.21–0.4 Fair, 0.41–0.6 Moderate, 0.61–0.8 Good and 0.81–1.0 Very good.

## Results

We used 212 sets of STIR/T1w FSE/T2w FSE sequences (125 subjects; mean-age 56 ± 18 years; 37 men, 49 unknown) and 2454 sets of T1w/T2w FSE and T1w-Dixon sequences (each sequence type fused to one 3D volume per subject and sequence; mean-age 53 ± 14 years; 1226 men, 2 others) for training. Testing was done on 36 (22 subjects; mean-age 62 ± 17 years; 6 men, 11 unknown) and 470 (mean-age 53 ± 13 years; 235 men) sequence pairs, respectively.

### Visual analysis

**Fig. 4:**
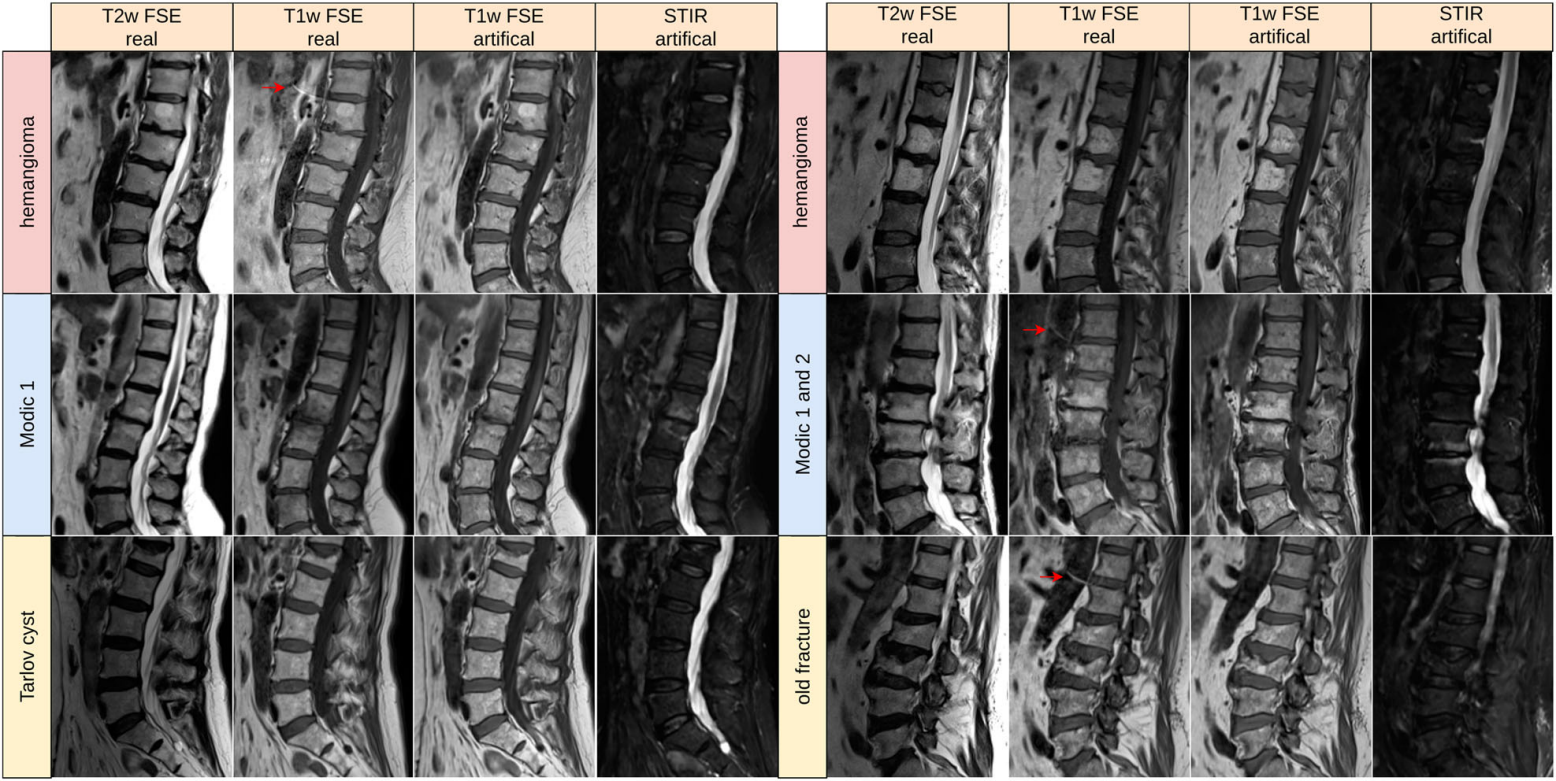
Examples from the Study of Health in Pomerania dataset with different anomalies. Artificial data was generated by the deep learning model with the T2w FSE and resampled axial T1-weighted gradient echo Dixon technique as input. Red arrows mark reconstruction artifacts in real T1w FSE data that are not reproduced in the translation. It is coincidental that we found multiple instances of these artifacts in a small sample set. STIR, sagittal short tau inversion recovery; T1w, sagittal T1-weighted image; T2w, sagittal T2-weighted; FSE, fast spin echo image

**Fig. 5:**
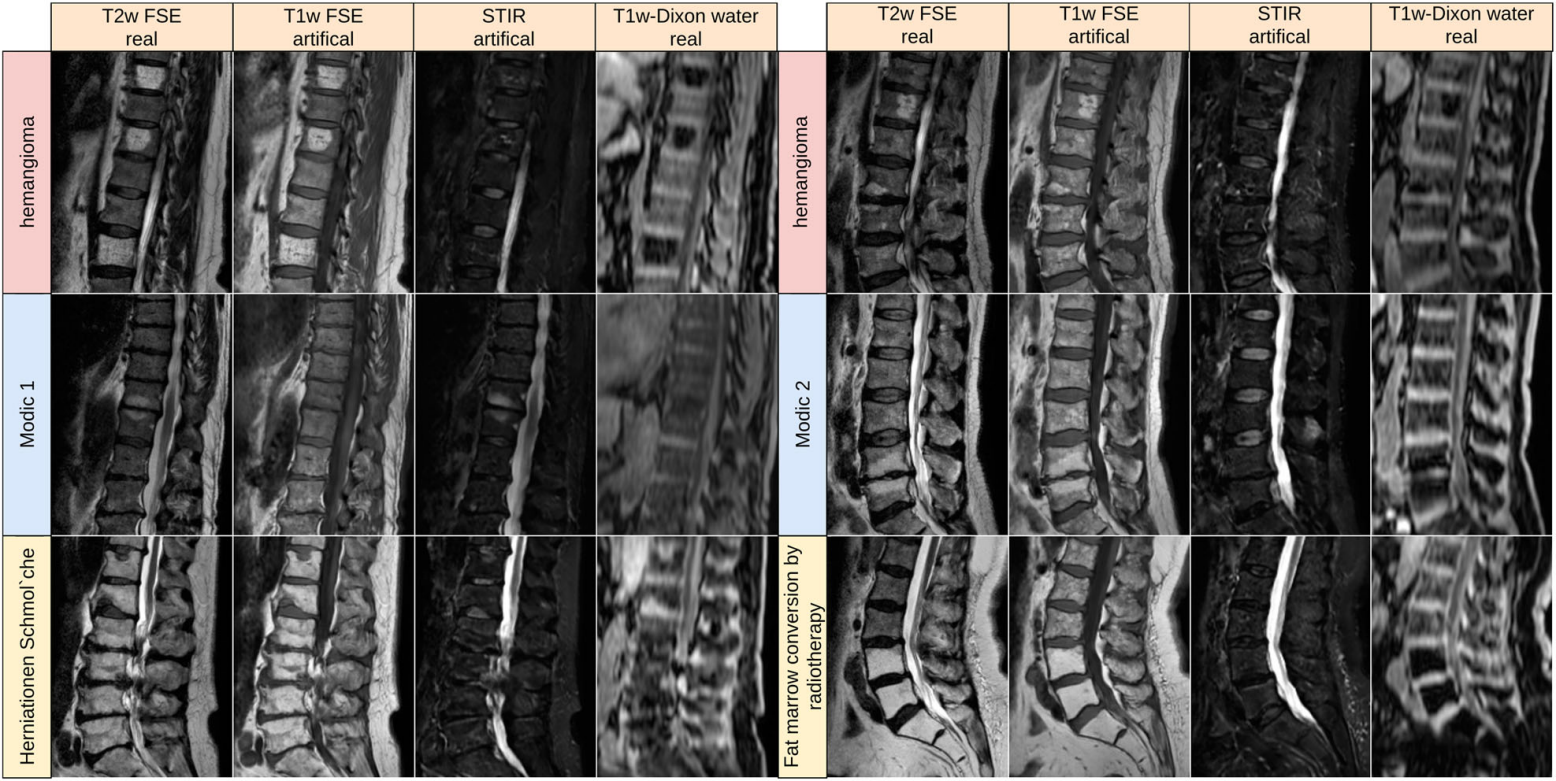
Examples of generated T1w FSE and STIR from the German National Cohort. Artificial data was generated by the deep learning model with the T2w FSE and resampled axial T1w-Dixon as input. We have no ground truth for this dataset. We can see that fatty aggregation causes black holes in the water image, but the look and feel of water image vary stronger than T1w FSE images. We show a variety of fatty anomalies like hemangioma, modic 2, fat marrow conversion and Herniationen Schmol’che. For modic 1, we see the signal drop in T1w FSE. STIR, sagittal short tau inversion recovery; T1w-Dixon water, axial T1-weighted gradient echo Dixon technique computed water part; T1w, sagittal T1-weighted image; T2w, sagittal T2-weighted; FSE, fast spin echo image

**Fig. 6:**
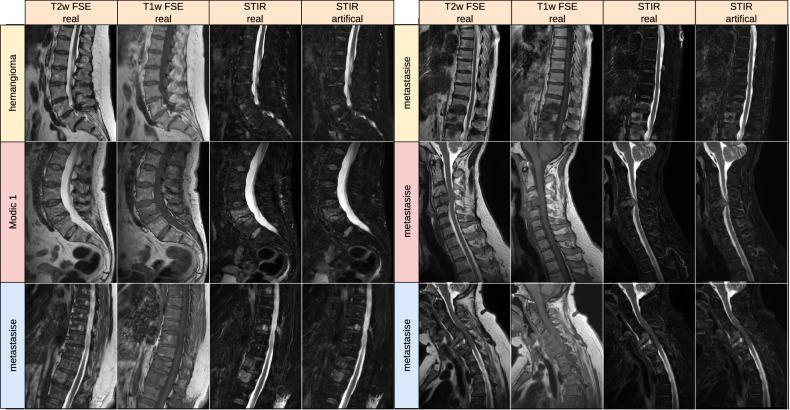
Examples of generated STIR from the Internal dataset. Artificial data was generated by the deep learning model with the T2w FSE and T1w FSE as input, as shown in previous studies. STIR, sagittal short tau inversion recovery; T1w, sagittal T1-weighted image; T2w, sagittal T2-weighted; FSE, fast spin echo image

In Fig. 4, we compare real and generated test samples of the SHIP dataset. The spine structure looks identical, while soft tissue has slightly moved between the two real acquisitions. The T1w FSE generation model is correct in translation water and fat hyperintensities. The spine images are nearly identical in real and artificial T1w FSE. Fatty degenerations like hemangioma and modic 2 are bright in both T1w and T2w FSE image. Watery aggregation like modic 1 and Tarlov cyst become dark in T1w FSE and bright in STIR, reproducing their expected signal behavior in these sequences. The SHIP dataset exclusively comprises old fractures. Thus the images lack the bright signals typically observed in STIR sequences, which would be characteristic of fresh fractures. In Figs. 5 and 6, we showcase pathologies like hemangioma and modic changes, where the generated images show the typical signal intensities. Even for rare or unseen pathologies during training, like fat marrow conversion by radiotherapy, the model correctly translates these tissues.

### Image quality

The tests to generate T1w FSE (Table 1) and STIR (Table 2) exhibit parallel outcomes. The unpaired generation (CycleGAN) performed worst. The final 3D approach had better results than its 2D counterparts. Not providing sagittal T1w FSE and axial T1w-Dixon has a negative impact on the translation quality. All *p*-values of the Wilcoxon signed-rank test between the proposed method and the others are < 0.01.

**Table 1:** Ablations on T1-weighted fast spin echo generation

Input		DL model	L1 ↓	MSE ↓	PSNR ↑	SSIM ↑
T2w FSE, T1w-Dixon	2D	CycleGAN	0.0142	0.0050	23.607	0.949
T2w FSE	2D	Pix2Pix	0.0100	0.0025	26.181	0.962
T2w FSE	3D	Pix2Pix	0.0157	0.0052	23.076	0.952
T2w FSE, T1w-Dixon	2D	Pix2Pix	0.0098	0.0025	26.192	0.961
T2w FSE, T1w-Dixon	3D	Pix2Pix	0.0089*	0.0021*	26.942*	0.965*

The values are computed from an authentic and a generated T1-weighted image from the same scan session. We compare whether an additional axial T1w-Dixon image, 2D or 3D and Pix2Pix (paired) or CycleGAN (unpaired) improve the translation. L1 and MSE: lower is better, and for PSNR and SSIM, higher is better. The best value is marked in bold with “*”

*L1* absolute error, *MSE* mean square error, *PSNR* peak signal-to-noise ratio, *SSIM* structural similarity image metric, *T1w-Dixon* axial T1-weighted gradient echo Dixon technique, *T1w* sagittal T1-weighted, *T2w* sagittal T2-weighted, *FSE* fast spin echo

**Table 2:** Ablations on sagittal short tau inversion recovery generation

Input			L1 ↓	MSE ↓	PSNR ↑	SSIM ↑
T2w FSE, T1w FSE	2D	CycleGAN	0.0144	0.0037	25.282	0.919
T2w FSE	2D	Pix2Pix	0.0113	0.0026	27.017	0.932
T2w FSE	3D	Pix2Pix	0.0159	0.0041	24.677	0.928
T2w FSE, T1w FSE	2D	Pix2Pix	0.0101	0.0020	28.234	0.940
T2w FSE, T1w FSE	3D	Pix2Pix	0.0095*	0.0018*	28.860*	0.948*

The values are computed from an authentic T1w-Dixon and a generated T1w FSE image from the same scan session. We compare whether an additional T1w FSE image, 2D or 3D and Pix2Pix (paired) or CycleGAN (unpaired) improve the translation. L1 and MSE: lower is better, and for PSNR and SSIM, higher is better. The best value is marked in bold with “*”

*L1* absolute error, *MSE* mean square error, *PSNR* peak signal-to-noise ratio, *SSIM* structural similarity image metric, *T1w-Dixon* axial T1-weighted gradient echo Dixon technique, *T1w* sagittal T1-weighted, *T2w* sagittal T2-weighted, *FSE* fast spin echo

### Turing test

The misclassification rate is 0.39 ± 0.09 for T1w FSE and 0.42 ± 0.18 for STIR. See Table 3 for individual results. The reviewers have a low interrater agreement with the Fleiss kappa test with 0.09 [−0.04 to 0.19–99% confidence interval] for T1w FSE and 0.09 [−0.10 to 0.13–99% confidence interval] for STIR images. For this test, a kappa below 0.2 is considered poor agreement and close to a random coin toss distribution.

**Table 3:** Misclassification rate of our seven reviewers (anonymized)

	T1w FSE	STIR
R1	0.38	0.56
R2	0.33	0.27
R3	0.41	0.52
R4	0.43	0.52
R5	0.28	0.25
R6	0.53	0.63
R7	0.39	0.21

For each column, 120 images with a natively acquired T2-weighted fast spin echo image and the potentially generated other modality are shown. Half of the image pairs are natively acquired, while the others are generated. The misclassification rate is the fraction of how many images were falsely labeled by the radiologist

*STIR* sagittal short tau inversion recovery, *T1w FSE* sagittal T1-weighted fast spin echo image

## Discussion

Generative AI models promise to replace missing sequences. Here, we developed and validated two generative models for sagittal T1-weighted fast spin echo (T1w FSE) and sagittal short tau inversion recovery (STIR) images from axial T1-weighted gradient echo Dixon technique (T1w-Dixon) and sagittal T2-weighted (T2w) FSE images of the spine. The axial T1w-Dixon has a lower sagittal resolution than our generated T1w FSE but still guides our network effectively in distinguishing fat and water in the T2w FSE image. Importantly, our artificial images faithfully recapitulate the imaging characteristics of these sequences and are indistinguishable from their real counterparts for trained neuroradiologists. Our models therefore offer unique opportunities to generate missing sequences in large epidemiological studies like the German National Cohort (NAKO) or “Study of Health in Pomerania” (SHIP).

This work extends earlier studies [1, 2], which generate STIR sequences from T1w FSE and T2w FSE inputs. Our ablation showed that the additional input of T1w-Dixon or T1w FSE image is necessary for the DL model. Otherwise, the model must guess necessary physical properties and extrapolate by looking at hyperintensities and whether water or fat is the cause. The general intention of multiple scans is to remove this ambiguity, and we should provide the DL application with this information to increase correctness. In our Turing test, neuroradiologists could not reliably differentiate real from artificial images. A misclassification of about 40% is consistent with other studies [2, 35]. During development, we had the impression that our STIR images were less susceptible to MR reconstruction artifacts and did not suffer from acquisition errors like real STIR. Our reviewers were blinded to this observation, which is reflected in the variance of our Turing test. Depending on the tendency of a rater to attribute acquisition errors toward machine learning might affect the misclassification rate. This would explain why some reviewers have good scores below 30% and others performed worse than random.

In contrast to previous studies [1, 2, 10–14], we utilized population-based data from the SHIP and NAKO rather than patient data with pathological changes. As a result, the pathology density in our training set was lower compared to other studies. We manually filtered pathologies for the NAKO test set to ensure an adequate number of pathological test samples for the Turing test. This data exhibited selection biases, as most pathologies were age-related non-acute cases, unlike the retrospectively collected internal data. The reviews were blinded to the varying likelihood of pathologies between real and generated images.

T1w-Dixon and STIR are specialized techniques, and due to time limitations, they are usually not produced in the same session. This limits this study because we have no data pairs with T2w, T1w-Dixon, and a STIR that would test our two networks from start to finish. We could only show a start to end in an unpaired manner, like the Turing test. The resolutions and MRI devices are different between the two trained networks. They should be generated on the same device with the same resolution for the sagittal images for optimal performance. We currently cannot compensate for patient movement.

Overall, we can generate artificial T1w FSE and STIR images retrospectively that can hardly be distinguished from real images. This enables us to generate the same data modalities we would encounter in everyday clinical practice in large biobank datasets.

## Abbreviations

FSE: Fast spin echo
GAN: Generative adversarial network
NAKO: German National Cohort (NAKO Gesundheitsstudie)
Pix2Pix: (Proper name)
PSNR: Peak signal-to-noise ratio
SHIP: Study of Health in Pomerania
SSIM: Structural similarity index measure
STIR: Short tau inversion recovery
T1w: T1-weighted
T1w-Dixon: Axial T1-weighted gradient echo Dixon technique; also, volume interpolated breath-hold examination
T2w: T2-weighted

## Footnotes

1 We made this software freely available under our GitHub: https://github.com/Hendrik-code/TPTBox/tree/main/TPTBox/stitching.
